# Supplementary material for: A new approach to the temporal significance of house orientations in European Early Neolithic settlements
Source: PLoS One. 2020 Jan 10;15(1):e0226082. doi: 10.1371/journal.pone.0226082 (PMC6953813; doi:10.1371/journal.pone.0226082)
Supplement: Attachment — Submitted filename: Mue-Schee_et_al_rebuttal.docx [file pone.0226082.s001.docx]

Answer to critique

Title (l. 1-2)

Both reviewers were unhappy with the title. On the one hand, it was deemed too specific (reviewer 1 + 2), on the other that it should be rephrased (reviewer 2). We followed both suggestions and rephrased the title completely.

Introduction (l. 32-75)

Reviewer 1 made the valuable suggestion to state in an additional introductory paragraph why the reader should be interested in the paper and to situate the study in time and space. We have added such a paragraph.

Conclusion (l. 393-414)

Reviewer 1 demanded that the conclusion should be really a conclusion and not an extension of the discussion, and that it should name additional avenues for testing the hypothesis. We have changed the conclusion accordingly.

Hypothesis „Change in orientation“

Reviewer 2 criticizes that „the entire argument of time-sensitivity of house orientation is not new“. We do not claim to have invented this hypothesis but on the contrary discuss the early suggestions by E. Sangmeister. But, in contrast to Sangmeister, we are able to present data to underpin the hypothesis.

Reviewer 2 states that s/he is not convinced by our line of argument at all. We would like to point out that obviously s/he misconceived some of our arguments. First and foremost, we do not deal with „the long-known phenomenon of the gradual westward - or eastward in eastern Europe - shift of Early Neolithic long houses accross Europe.“ We only look at change in orientation PER SITE (or small scale region, in the case of the Merzbachtal).

We completely agree that „the regional variation [is] considerable“ but in our view the question why this is the case is a completely different topic. Local studies explicating „jumps“ in orientation concern, for example, the surrounding of Dresden (Link Th [2011] Böhmische Dörfer? Zur Stellung der Dresdener Elbtalweitung zwischen sächsischer und böhmischer Bandkeramik. In Th. Doppler, B. Ramminger, D. Schimmelpfennig (eds.) Grenzen und Grenzräume? Kerpen-Loogh: 11-24) or Southern Bavaria (Pechtl J [2010] Anmerkungen zum Kenntnisstand linienbandkeramischer Hausarchitektur im südöstlichen Bayern und zum Potenzial ihrer typologischen Auswertungen. Fines Transire 19: 35-51), and in our view such „jumps“ are highly significant, but beyond the scope of the present paper.

It has been claimed again and again that the variation in orientation follows „local topography“ (e. g., because of prevailing currents). However, where this suggestion was put to the test (Mattheußer E (1991) Die geographische Ausrichtung bandkeramischer Häuser. In: Studien zur Siedlungsarchäologie 1. Bonn: Habelt. pp. 1-49), it turned out to be not valid. Anyway, even if true, it would not explain variation per site.

We would be keen to hear of the „some sites [where] we can observe shifts in west-ward [= clockwise?] orientation which is a counterargument against gradual visual misconception.“ We actually looked at a much larger number of sites than presented in the paper but only found the same tendency of counter-clockwise shifts. Bylany may be the only possible exception when it comes to major excavated sites. Unfortunately, the available data is presented in such a condensed manner (Pamatky archeologicke 77, 1986, 397 Table 49) that it is difficult to draw definite conclusions.

The point is, and this is clearly stated in text, that the pseudoneglect explanation only is relevant where there are no other reasons (topography, agglomeration of houses, circular arrangement of houses). There might also be other factors we have not accounted for, or that we archaeologically cannot account for. However, the evidence of counter-clockwise shifts is strong, and it is clearly not to be dismissed even if there were singular occurences of clockwise ones.

To account for the critique of reviewer 2, we added explaining sentences at the beginning, in the middel and at the end.
